# Associations between Gun Ownership and Firearm Homicide Rates in US States

**DOI:** 10.1007/s11524-023-00734-x

**Published:** 2023-06-29

**Authors:** Konstantinos Christopoulos

**Affiliations:** University of Piraeus, 80 Karaoli & Dimitriou Street, Piraeus, 185 34 Greece

**Keywords:** Bayesian statistics, Gun violence, Homicide, Firearm deaths, United States

## Abstract

The United States combine high rates of firearm homicides with high gun prevalence. In the past, a significant positive association was found between the two. This study revisits the gun prevalence-gun homicide debate using more elaborate estimates of gun ownership for the 50 States. Longitudinal data (1999–2016) were analysed with Bayesian multilevel Gamma-Poisson models. The results demonstrated a very small positive association that diminished after adjusting for crime rates. Findings suggest that the association either attenuated in more recent years, or previous studies had overestimated this association.

## Introduction

News of firearm homicides have become a daily reality in the US. This of course comes as no surprise since US has very high household ownership levels of private firearms, the weakest gun control laws, and the highest homicide rates [1]. Despite geographic variation, the prevalence of gun owners in the majority of the States remains higher than most of the developed world [2].

Firearms contribute to mortality via assault, suicide, and unintentional deaths (accidents). While much of the literature has focused on firearm suicides, this study focuses on assault mortality in the US. Earlier studies, including case–control, cohort, and ecological studies, more or less agree that higher levels of gun prevalence increase firearm homicide rates. A positive association was found for male, female and child firearm deaths [3–5], as well as for homicides in general [6]. Hepburn and Hemenway [1] provide a review on these earlier studies.

In more recent years, US studies found a weaker association with firearm homicides and a stronger positive association with firearm suicides [7]. The association nevertheless remains positive as Siegel et al. [8], who used US state longitudinal data from 1981 to 2010, found. This study uses both gun ownership proxies and survey-derived measures of household gun ownership and deals well with confounding factors. The results were also robust to different model specifications. In a consequent analysis, Siegel et al. [9] divided the sample into stranger and non-stranger homicides, but found a significant association *only* with non-stranger homicide rates.

Most of ecological studies use the percentage of suicides committed with a firearm—an instrument that shows high validity [10]—as a measure of gun prevalence. On the other hand, measurement on the individual-level comes from various surveys such as the General Social Survey (GSS), the Behavioral Risk Factor Survey (BRFSS), and the Small Arms Survey. The absence of better estimates for ecological studies makes inference tentative even when confounding has been adequately tackled.

This study aims to explore the association between gun ownership and firearm mortality rates from assaults at the US state level. To this end, longitudinal data for the years 1999–2016 and a Bayesian specification were employed. The research contributes to the literature by 1) using the most recent time-series to date and 2) using estimates for gun ownership that take into account all previously used proxies. Results demonstrate that the association might have been overestimated in previous studies.

## Material & Methods

### Data

The data cover the 50 US states for the years 1999–2016. The annual series does not extend any further due to the unavailability of more recent gun ownership estimates. Data for firearm mortality and population size were extracted from the Centers for Disease Control and Prevention (CDC) WONDER underlying cause of death database [11] using the ICD-10 codes X93 (Assault by gun discharge), X94 (Assault by rifle, shotgun and larger firearm discharge), and X95 (Assault by other and unspecified firearm discharge). Crime rates were retrieved from the Federal Bureau of Investigations [12].

Gun ownership estimates were retrieved from the RAND corporation [13]. The estimates concern the proportion of adult, non-institutionalised residents who live in a household with a firearm. These data have the advantage of including several variables—which are commonly used as a proxy for gun ownership—for the estimation of the proportion of gun owners, including: various survey-based estimates, administrative data on firearm suicides, permits to purchase, background checks, hunting licenses, and subscriptions to the Guns & Ammo magazine. This fact, combined with the structural equation modelling for the estimation, provides perhaps the best available ecological measurement of this exposure. Table 1 offers some basic descriptive statistics for the variables used in the analysis.

**Table 1:** Descriptive statistics for firearm mortality, gun ownership, and crime rates ($N=900$)

	*Median*	*IQR*	*Min.*	*Max.*
*Firearm mortality*	123	342–30	0	1883
*Firearm mortality rates (per 100,000)*	3.18	4.74–1.60	0	11.24
*Gun ownership (%)*	40	48–34	3	69
*Violent crime rates (per 100,000)*	353	500–263	70	854
*Property crime rates (per 100,000)*	3016	3100–2471	1407	5833

*IQR* Interquartile range ($Q_{3}$–$Q_{1}$)

### Statistical Analysis

#### Bayesian Methods

Firearm mortality counts were analysed with Gamma-Poisson models using Hamiltonian Monte Carlo (HMC) sampling as implemented in *Stan* version 2.31. Weakly informative priors were preferred. Markov Chain convergence and sampling were inspected with traceplots and ranked traceplots, as well as with $\hat{R}$ and *Stan*’s estimate of effective number of samples. HMC’s divergent transitions warnings were also taken into account.

The results are presented graphically with posterior distribution densities and posterior predictive simulations, as well as with posterior means and 89% Highest Posterior Density Intervals (HPDI). The analysis was performed using the *R* package ‘*rethinking*’. For more details on the Bayesian methods used in this study we refer the reader to McElreath [14].

#### Identification Strategy

**Fig. 1:**
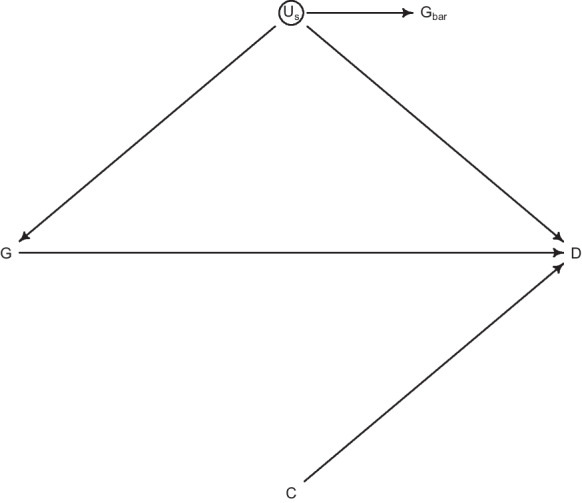
Directed Acyclic Graph. Notes: G=gun ownership; D=firearm deaths; C=crime rates; U$_{s}$=Unobserved State confounds; G$_{bar}$=State gun ownership mean. The DAG was created using the *R* package ‘*dagitty*’

Two models were used in the analysis. A Mundlak model (MM) [15] and a fixed-effects (FE) model. While both models have the ability to adjust for the time-invariant characteristics of the States, the FE model does a better job at this. The MM on the other hand takes advantage of the partial pooling, since it is a random-effects model which includes the State mean of the exposure (gun ownership) as a covariate in order to adjust for the time-invariant characteristics of States. It does so because the State-mean of the exposure is a descendant of the unobserved State-level confounds, as is show in Fig. 1, and therefore shares variation with those unobserved characteristics.

Aside from adjusting for time-invariant cultural and societal factors associated with gun ownership and the lethality of crimes, the models were augmented with crime rate variables, namely violent and property crime rates, as well as their interaction, as predictors and potential confounders.^1^ The two models are presented next.

#### Statistical Models

The Gamma-Poisson Mundlak model for firearm deaths (*D*) is the following:

$$\begin{aligned} D\sim Gamma-Poisson(\lambda , \phi ) \end{aligned}$$

1 $$\begin{aligned} log\ \lambda _i\!=\! & {} \log \ \pi _{i} \!+\! \alpha _{State[j]} + \beta _{1}G_i + \beta _{2}VC_i \!+\! \beta _{3}PC_i \nonumber \\{} & {} \quad + \beta _{4}VC_i\times PC_i + \beta _{5}\bar{G}_{State[j]} \end{aligned}$$

where $log\ \pi _{i}$ is the population offset, $\alpha _{State[j]}$ is the random intercept, $G_i$ is the standardised percentage of gun ownership, and $VC_i$ and $PC_i$ are the standardised crime rates per 100,000 inhabitants for violent and property crimes, respectively. $\bar{G}_{State[j]}$ is the mean gun ownership for each State. The priors used were:

$$\begin{aligned} \alpha _{State}\sim Normal(\bar{\alpha },\tau ) \end{aligned}$$

$$\begin{aligned} \bar{\alpha }\sim Normal(-10,1) \end{aligned}$$

$$\begin{aligned} \tau \sim Exponential(1) \end{aligned}$$

$$\begin{aligned} \beta _{1,2,3,4,5}\sim Normal(0,1) \end{aligned}$$

The Gamma-Poisson Fixed-effects model is the following:

$$\begin{aligned} D\sim Gamma-Poisson(\lambda , \phi ) \end{aligned}$$

2 $$\begin{aligned} \log \ \lambda _i\!=\! & {} \log \ \pi _{i} \!+\! \alpha _{State[j]} \!+\! \beta _{1}G_i \!+\! \beta _{2}VC_i \!+\! \beta _{3}PC_i\nonumber \\{} & {} \quad + \beta _{4}VC_i\times PC_i \end{aligned}$$

where $\alpha _{State[j]}$ are now the State fixed-effects with prior

$$\begin{aligned} \alpha _{State}\sim Normal(-10,1) \end{aligned}$$

## Results

**Fig. 2:**
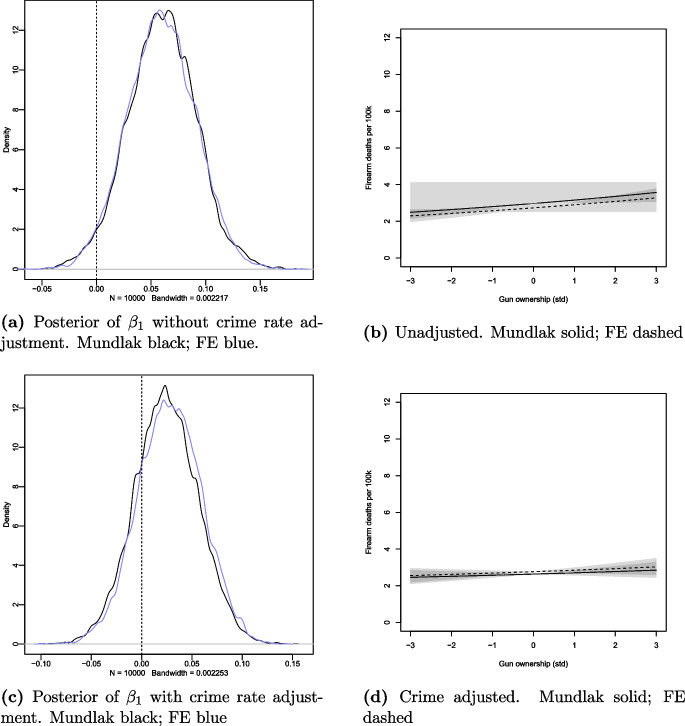
Posterior distributions of gun ownership coefficients (a, c) and posterior predictive simulations from Mundlak and fixed-effects models (b, d)

Since the exposure in question was standardised, for better comprehension, the standard deviation of gun ownership is approximately 13.6%. For reasons discussed later in the paper, we first present the model estimates without crime rate adjustments. The MM and FE models yielded the same results with posterior mean 0.06, Standard Error (SE) 0.03, and 89% HPDI 0.01–0.11. Figure 2a shows the posterior density and Fig. 2b the posterior predictive simulation for the two models.

**Fig. 3:**
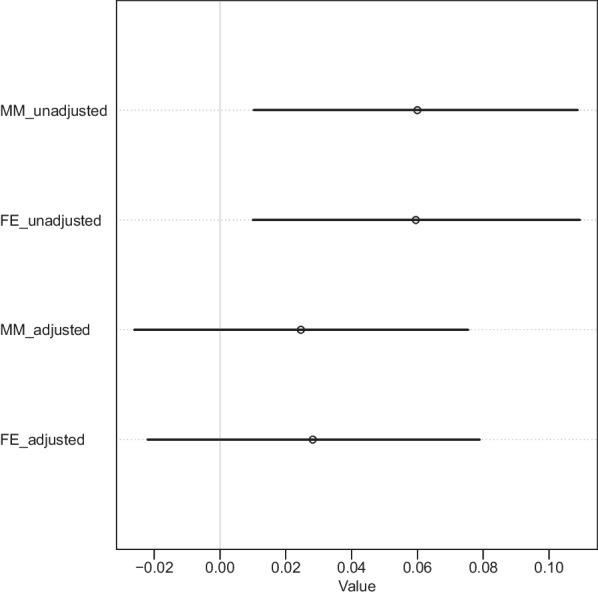
Forest plot for the gun ownership coefficients and 89% HPDI

This small positive association was attenuated when the crime rate adjustments were introduced to the models. The results for MM and FE were very similar with posterior mean 0.02, SE 0.03, and 89% HPDI $-$0.02–0.08; and posterior mean 0.03, SE 0.03, and 89% HPDI $-$0.02–0.08, respectively. Figures 2c and d show the corresponding posterior densities and predictive simulations for the two models, respectively. Figure 3 summarises the findings.

## Discussion

This study aimed at examining the association between gun ownership and gun-related mortality across US states. While adjusting for State-level confounding showed a small positive association, this association diminished after controlling for violent and property crimes rates.

Our estimates for incidence rate ratio (IRR), namely 6% and 3% (obtained by exponentiating the posterior means) for a one SD increase (13.6%), are much lower than the cross-sectional survey estimate of Miller et al. [5], which was a 47.1% increase in firearm homicide rates (SD=12%). Compared with other studies that used similar methods and data to ours, such as Siegel et al. [8], the difference was smaller—12.9% firearm homicide rate increase for a 13.8% increase in gun ownership. The latter study uses the percentage of suicides committed with a firearm as a proxy for gun prevalence, therefore is it unclear whether the difference in estimates comes from the more recent time-series, the measurement of the exposure, or residual confounding.

This is yet another study that debunks the idea that more guns will lead to less gun violence and therefore less deaths by firearms—although not in an emphatic way as its predecessors. It becomes apparent that as time progresses the potential effect of gun ownership on firearm-related deaths diminishes. This fact, combined with the perpetuation of gun violence incidents, points the finger to other societal factors that may lead to these behaviours, such as the social capital [16]. Studying the interaction between these factors and gun ownership might shed some light into why we observe a close to null effect.

The following three paragraphs outline the limitations of this study. First, the analysis is performed on an ecological level, namely the US states, and is therefore prone to an ecological fallacy. A more disaggregated unit of analysis or individual level data may produce different results. The transportability of the findings outside the US is also limited. Secondly, the bidirectional causality scenario, i.e., firearm mortality rates also causing gun ownership [17], cannot be ruled out despite evidence against it [8].

Residual confounding is always present in observational studies. Although the MM and FE models deal with State-level confounding, they do nothing for time-varying confounders. The inclusion of the crime rates aimed—aside from the necessity to include good predictors in generalised linear models—at adjusting for factors such as racial composition, urbanisation, unemployment, and inequality, which influence crime rates (they are antecedents of C), and consequently firearm mortality. An arrow from crime rates toward gun ownership was not added in Fig. 1 since crime rates did not have a significant correlation in our data—contrary to the findings of Monuteaux et al. [18]. This points to the fact that, at least in our data, crime rates are not mediators, that is, influenced by gun ownership (positively or negatively) and sequentially affecting firearm mortality. A review by Kleck [19] on the matter also comes to the same conclusion. Nor are they confounders—in the case where owning guns for protection is associated with local crime rates—but instead independent predictors of the outcome.

Despite best efforts in exposure measurement, some degree of measurement error is unavoidable with estimates. Regardless of measurement, gun ownership is not an ideal measure of firearm availability since it does not provide information on the number, lethality, and concealability of the guns, nor on the feasibility of obtaining guns within States, or from adjacent States with less restrictions [1, 20].

In conclusion, our study did not find a significant association between gun ownership and gun-related deaths at the US state level. While a very small positive association might exist, the findings clearly reject the hypothesis that gun ownership lowers homicide rates. Future research may want to focus on effect modifiers that are of potential interest for prevention and public health policy.

## Data Availability

The code used for the analysis is available on https://github.com/KonstantinosChristopoulos/Christopoulos-JoUrbanHealth-2023. Data cannot be shared due to CDC’s data confidentially restrictions but will be made available upon reasonable request.

## Footnotes

1 Violent crime rates include the offences of murder, rape, robbery, and aggravated assault. Property crime rates include the offences of burglary, larceny-theft, and motor vehicle theft.
